# Hapten designs based on aldicarb for the development of a colloidal gold immunochromatographic quantitative test strip

**DOI:** 10.3389/fnut.2022.976284

**Published:** 2022-08-23

**Authors:** Hong Shen, Yuping Wan, Xiaosheng Wu, Yu Zhang, Jingwen Li, Tingting Cui, Han Sun, Haifeng Cui, Kailun He, Guangpeng Hui, Xu Chen, Guoqiang Liu, Meihong Du

**Affiliations:** ^1^Biological Inspection Department, Zhejiang Institute for Food and Drug Control, Hangzhou, China; ^2^Beijing Kwinbon Biotechnology Co., Ltd., Beijing, China; ^3^Beijing Engineering Research Centre of Food Safety Immunodetection, Beijing, China; ^4^Beijing Center for Physical and Chemical Analysis, Institute of Analysis and Testing, Beijing Academy of Science and Technology, Beijing, China

**Keywords:** aldicarb, hapten, monoclonal antibody, immunoassay, colloidal gold immunochromatography, test strip

## Abstract

The common carbamate insecticide aldicarb is considered one of the most acutely toxic pesticides. Herein, rational design was used to synthesize two haptens with spacers of different carbon chain lengths. The haptens were then used to immunize mice. The antibodies obtained were evaluated systematically, and a colloidal gold immunochromatographic strip was developed based on an anti-aldicarb monoclonal antibody. The 50% inhibition concentration and linear range of anti-aldicarb monoclonal antibody immunized with Hapten 1 were 0.432 ng/mL and 0.106–1.757 ng/mL, respectively. The cross-reactivities for analogs of aldicarb were all <1%. The limit of detection of the colloidal gold immunochromatographic strip was 30 μg/kg, and the average recoveries of aldicarb ranged from 80.4 to 110.5% in spiked samples. In the analysis of spiked samples, the test strip could accurately identify positive samples detected by the instrumental method in the GB 23200.112-2018 standard but produced some false positives for negative samples. This assay provides a rapid and accurate preliminary screening method for the determination of aldicarb in agricultural products and environments.

## Introduction

Aldicarb, chemically known as [(*E*)-(2-methyl-2-methylsulfanylpropylidene)amino]*N*-methylcarbamate, is a carbamate insecticidal, acaricidal, and anti-nematodal compound that is widely used to treat cotton, peanut, corn, and many other crops (1). Although aldicarb is easily degraded, with a half-life of only 5–12 days, degradation products such as aldicarb sulfoxide and aldicarb sulfone have stronger water solubility and environmental toxicity than the parent substance, leading to long-term pollution after dissolution in water (2). Therefore, they can often be detected in soil, air, water, and agricultural products (3–6), which has attracted extensive attention in various countries. For example, in China, the maximum residue limit (MRL) for aldicarb in bulb vegetables, brassica vegetables, and leaf vegetables is 0.03 mg/kg (7). Japan and England have set MRLs of 0.01–0.5 mg/kg for aldicarb in crops and vegetables. Therefore, it is necessary to develop sensitive, accurate, and efficient detection methods for aldicarb in agricultural products and environments.

At present, various instrumental analysis methods, such as gas chromatography and high-performance liquid chromatography–mass spectrometry (HPLC–MS), have been widely used for the detection of aldicarb (8–10). These methods are highly sensitive and provide accurate quantification and simultaneous detection of multiple indicators. However, these methods require expensive instruments, ongoing maintenance, professional technicians, cumbersome sample processing, and long detection times and, furthermore, are unsuitable for on-site detection (11). Immunological techniques are simple, economical, and rapid and thus can circumvent the shortcomings of instrumental methods (12). However, because aldicarb is toxic and unstable in the environment, there are few studies on haptens based on aldicarb. The first aldicarb hapten and associated enzyme-linked immunosorbent assay (ELISA) were developed in 1988 by Brady et al. (13). The aldicarb oxime hapten was prepared from *trans*-(aminomethyl)-cyclohexanecarboxylate, had high selectivity and was detectable at a low level of 0.3 mg/kg. In 2003, Siew et al. (14) synthesized a hapten based on aldicarb oxime ethyl acetate and coupled it with the carrier proteins bovine serum albumin (BSA) and keyhole limpet hemocyanin (KLH) to form two kinds of immunogens. They obtained two different monoclonal antibodies, but their inhibitory activities were not high (IC_50_ = 200 ng/mL), and the detection limit of the national standard was not achieved. Zhang et al. (15) designed and synthesized a new aldicarb hapten, aldicarb oxime succinic ester, and coupled it with BSA to prepare a polyclonal antibody. However, further research using an ELISA was not carried out, and the corresponding monoclonal antibody was not obtained. Yao (16) used aldicarb oxime as a raw material in one-step and two-step reactions to prepare two aldicarb haptens, which were then used to produce a monoclonal antibody with an IC_50_ of 18.505 ng/mL. Dichloromethane, which is a highly toxic solvent with a low boiling point, was used in the costly preparation process to purify the product (17). Although these antibodies have been used for the detection of actual samples, they all have certain limitations, and for samples with complex matrices, they need to be diluted substantially to exclude matrix interference. Liu et al. (18) developed a rapid, simple, and sensitive immunochromatographic strip test to detect aldicarb in cucumber samples. The cutoff limit of the test strip for aldicarb was 100 ng/mL; however, this did not meet the MRL specified in the GB 2763 standard (7). Therefore, it is necessary to further improve the sensitivity of immunoassays using aldicarb haptens.

An effective immunoassay method requires antibodies with high affinity and selectivity. To achieve this, a suitable hapten for the immunogen is needed. Aldicarb has a small relative molecular mass and no immunogenicity, and thus it must be conjugated to a macromolecular carrier with immunogenicity (19). To realize coupling to macromolecular substances, the aldicarb hapten molecule must have an active group (such as –NH_2_, –COOH, –OH, and –SH) that can covalently bind to the carrier, and to enhance the performance of the resulting antibody, the spacer arms of the hapten should be of a certain length (20). Current studies have found that the length and composition of the spacer arm affect the ability of the antibody to recognize the analyte, that is, the affinity of the antibody for the analyte, because the spacer arm affects the properties and structures of small pesticide molecules in artificial antigen molecules (21–23).

In this study, two aldicarb haptens differing in the carbon chain length of the spacer arm were used to prepare highly specific and highly sensitive anti-aldicarb monoclonal antibodies with an IC_50_ of 0.432 ng/mL. Compared with other antibodies (13–16, 18), the prepared monoclonal antibodies have significantly improved sensitivities. The immunological properties of the two antibodies were compared. In addition, a simple, rapid colloidal gold immunochromatographic method was developed, and its sensitivity and accuracy were evaluated. Specifically, spiked samples were analyzed using both the immunochromatographic method and an instrumental method, and the results from both methods were statistically compared.

## Materials and methods

### Reagents and apparatus

BALB/c mice (license number: SCXK2019-0010) were procured from SPF Biotechnology Co., Ltd. (Beijing, China). Standard materials (aldicarb, aldicarb sulfone, aldicarb sulfoxide, thiofanox, oxamyl, methomyl, metolcarb, isoprocarb, and carbofuran) were acquired from Dr. Ehrenstorfer GmbH (Augsberg, Germany). Freund’s complete adjuvant and Freund’s incomplete adjuvant, phosphate buffered saline (PBS; 10×), KLH, ovalbumin (OVA), *N*,*N*-dimethylformamide (DMF), 1-ethyl-(3-dimethylaminopropyl) carbodiimide (EDC), *N*-hydroxysuccinimide (NHS), and goat anti-mouse IgG secondary antibody were purchased from Sangon Biotech Co., Ltd. (Shanghai, China). A mouse monoclonal antibody subtype identification ELISA kit was purchased from Sino Biological Co., Ltd. (Beijing, China).

UPLC–MS (Acquity UPLC, Waters, Milford, MA, United States and QTRAP 4000, SCIEX, Framingham, MA, United States) was used to identify the hapten structure. Hydrogen nuclear magnetic resonance (^1^H NMR; AVANCE III-600, Bruker, Germany) spectra were also obtained to identify the hapten structure. Ultraviolet–visible (UV–Vis; UV-3600, Shimadzu, Japan) spectra were used to verify the coupling of haptens to carrier proteins. Other equipment included a GT-810 colloidal gold reader (Beijing Kwinbon Biotechnology Co., Ltd., Beijing, China).

### Preparation of aldicarb hapten

**FIGURE 1:**
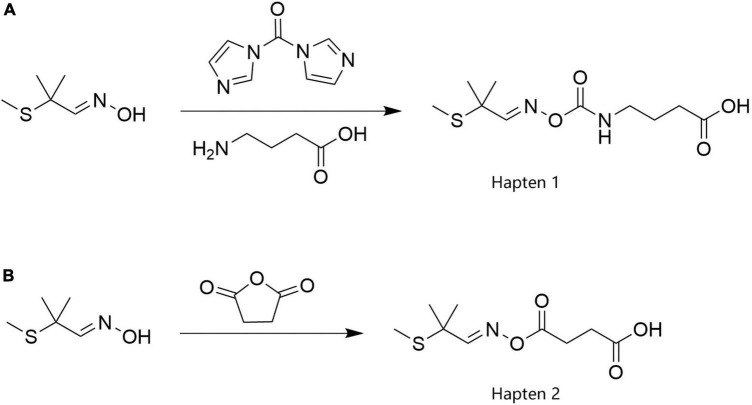
The synthesis routes of aldicarb haptens.

Because the length and composition of the spacer arm in the hapten structure determine the specificity and sensitivity of the resultant monoclonal antibodies, in this study, spacer arms with different carbon chain lengths at the same position on the aldicarb molecule were used to prepare two haptens (Hapten 1 and Hapten 2). The synthetic processes are illustrated in Figure 1. The MS and ^1^H NMR data for these haptens are shown in Supplementary Figure 1.

2-Methyl-2-(methylsulfanyl)propanaldoxime (1.33 g) was dissolved in 20 mL of DMF, to which 1.94 g of *N*,*N*-carbonyldiimidazole was added. The mixture was then stirred, and the reaction occurred at room temperature for 2 h. Then, 5 mL of an aqueous solution containing 2.06 g of aminobutyric acid was added, and the mixture was stirred at room temperature for 4 h. After the reaction was completed, 200 mL of water was added, and the mixture was stirred and transferred to a separatory funnel. Then, 200 mL of ethyl acetate was added, and the mixture was shaken and allowed to stand still. The aqueous phase was separated, and the organic phase was evaporated to dryness to obtain a yellow oily substance, which was passed through a silica gel column (petroleum ether: ethyl acetate = 5:1). Finally, 1.72 g of the carboxy-aldicarb hapten product, Hapten 1, was obtained, and the yield was 65.65%. The product was characterized by ESI-MS and ^1^H NMR. ESI-MS, *m*/*z*: 261.16 [M–H]^–^; ^1^H NMR (600 M*Hz*, DMSO): δ12.07 (s, 1H), 7.62 (s, 1H), 3.37 (s, 5H), 3.08 (dd, *J* = 13.0, 6.6 *Hz*, 2H), 2.53–2.47 (m, 2H), 2.23 (t, *J* = 7.4 *Hz*, 2H), 1.94 (s, 3H), 1.67 (p, *J* = 7.2 *Hz*, 2H), 1.40 (s, 6H).

2-Methyl-2-(methylsulfanyl)propanaldoxime (1.33 g) was dissolved in 50 mL of pyridine, to which 1.29 g of succinic anhydride was added. The mixture was stirred, heated to 80^°^C, and allowed to react for 4 h. After the reaction was completed, the mixture was cooled to room temperature, and pyridine was removed by rotary evaporation. The residue was dissolved in 200 mL of water and 8 mL of 1 M hydrochloric acid, and the pH of the mixture was adjusted to 6. Then, 150 mL of ethyl acetate was added, and the mixture was stirred and transferred to a separatory funnel. The aqueous phase was separated, and the organic phase was evaporated to dryness by rotary evaporation to obtain a crude product, which was passed through a silica gel column (dichloromethane:methanol = 10:1) to obtain 2.12 g of the aldicarb succinate hapten product, Hapten 2, with a yield of 90.99%. The product structure was confirmed by ESI-MS and ^1^H NMR. ESI-MS, *m*/*z*: 232.26 [M–H]^–^; ^1^H NMR (600 M*Hz*, DMSO): δ12.23 (s, 1H), 7.75 (s, 1H), 3.33 (s, 12H), 2.62 (dd, *J* = 7.4, 5.5 *Hz*, 2H), 2.52 (dd, *J* = 7.5, 5.5 *Hz*, 2H), 2.51–2.49 (m, 5H), 1.95 (s, 3H), 1.40 (s, 6H).

### Preparation of artificial antigens

Using the active ester method (EDC/NHS) (24), the synthetized haptens were coupled to the carrier proteins KLH and OVA.

To synthesize the first immunogen, 1.74 mg of Hapten 1 was dissolved in 0.3 mL of DMF, to which 3.06 mg of NHS and 5.01 mg of EDC were added. The mixture was stirred at room temperature for 2 h. Then, 10 mg of KLH was dissolved in 1 mL of carbonate–bicarbonate (CB) buffer (0.1 M, pH 9.1), and the hapten reaction solution was slowly added. The mixture was stirred at room temperature for 4 h. After the reaction was completed, the mixture was placed into a dialysis bag for dialysis with 0.02 M PBS for 3 days. The medium was changed three times a day. The dialyzed mixture was centrifuged to obtain the immunogen Hapten 1-KLH, which was stored at –20°C. To synthesize the second immunogen, 1.54 mg of Hapten 2 was dissolved in 0.3 mL of DMF, to which 4.54 mg of NHS and 7.6 mg of EDC were added. The remaining steps were the same as the above operation, which was applied to obtain the immunogen Hapten 2-KLH.

To synthesize the coated hapten, Hapten 1 (11.65 mg) was dissolved in 1 mL of DMF, to which 21 mg of NHS and 35 mg of EDC were added. The mixture was stirred at room temperature for 2 h. Then, OVA (100 mg) was dissolved in 1 mL of CB buffer (0.1 M, pH 9.1). The remaining steps were the same as the above operations, which were applied to obtain Hapten 1-OVA. Similarly, to synthesize the second coated hapten, Hapten 2 (10.3 mg) was dissolved in 1 mL of DMF, to which 20.4 mg of NHS and 33.1 mg of EDC were added. The remaining steps were the same as the above operations, which were applied to obtain Hapten 2-OVA.

The aldicarb haptens, carrier proteins, and conjugates synthesized above were examined using UV spectrophotometry to determine whether the hapten was successfully coupled to the carrier protein.

### Preparation and titer detection of antiserum

Healthy 6–8-week-old female BALB/c mice were selected and immunized with the immunogens Hapten 1-KLH and Hapten 2-KLH at a dose of 200 μg. For the first immunization, an equal volume of Freund’s adjuvant was used for emulsification. After emulsification was completed, subcutaneous injection was carried out on the back of the neck at multiple points. Thereafter, the dose was boosted and emulsified with Freund’s incomplete adjuvant, and the mice were immunized once every 14 days for a total of 3 immunizations. On the seventh day after the fourth immunization, the blood of the mice was taken from the severed tail and centrifuged at 5,000 r/min for 5 min to obtain mouse serum. Serum titers were determined by indirect ELISA (25), and the spleen was selected for subsequent experiments.

### Preparation of monoclonal antibodies and analysis of their immunological properties

Mouse spleen cells were fused with myeloma cells, and the cell lines that could stably secrete anti-aldicarb monoclonal antibodies were screened through cell culturing, screening, and other steps (26). Ascites antibodies were prepared by *in vivo* induction, and the anti-aldicarb monoclonal antibody was purified using octanoic acid and saturated sulfuric acid following the ammonium method (27).

A mouse monoclonal antibody subtype identification ELISA kit was used to identify the antibody subtype. Indirect competitive ELISA (icELISA) was used to determine the 50% inhibitory mass concentration (IC_50_) of the monoclonal antibody against aldicarb, and IC_50_ was used to measure the sensitivity of the monoclonal antibody (28). Cross-reaction tests were used to determine specificity, in which aldicarb sulfone, aldicarb sulfoxide, thiofanox, oxamyl, methomyl, metolcarb, isoprocarb, and carbofuran were selected as inhibitors. The IC_50_ of each inhibitor was determined by icELISA, and the IC_50_ of the monoclonal antibody against aldicarb and the IC_50_ of the inhibitor were taken as the cross-reaction rate.

### Sample preparation

Cabbage and leek samples obtained from local markets were confirmed to be negative for aldicarb using liquid chromatography (LC). The parameters for LC analysis are described in supplementary section 1.

The negative samples were mixed with an aldicarb standard solution to make positive cabbage and leek samples with aldicarb concentrations of 30 and 60 μg/kg, respectively. Each sample was extracted according to the following method. The cabbage and leeks were cut into 1-cm square pieces. The sample (2.00 ± 0.05 g) was mixed with 6 mL phosphate buffer (0.1 M, pH = 7.4), and the mixture was vortexed for 1 min and allowed to stand for 5 min. The upper liquid layer was tested.

### Preparation of colloidal gold immunochromatographic test strips

Colloidal gold particles were prepared following the trisodium citrate reduction method (29), and the surface of the gold nanoparticles was labeled with the anti-aldicarb monoclonal antibody by referring to the method of Liu (30). Thereafter, 100 μL of anti-aldicarb monoclonal antibody-labeled colloidal gold was added to the microplate. The microplate was then treated for 3 h in a cold trap at –50°C and vacuum-dried for 15 h to obtain a lyophilized microporous reagent with anti-aldicarb monoclonal antibody-labeled colloidal gold, which was sealed.

The nitrocellulose (NC) membrane was coated with Hapten 1-OVA (1 mg/mL) as the test line (T line) and goat anti-mouse IgG antibody (0.5 mg/mL) as the control line (C line). The coated NC membrane was dried at 37°C for 16 h. The sample pad was immersed in working buffer for 2 h and dried at 37°C for 2 h.

The NC membrane coated with capture reagents was pasted in the center of the PVC backing plate and the sample pad, and the absorbent pad was laminated and pasted onto the backing plate. Finally, the plate was cut into 4-mm-wide strips.

### Optimization of the test strip

The type of NC membrane and sample pad and the working buffer for the sample pad were optimized to improve the sensitivity of the immunoassay. The immunoassays were evaluated using the coefficient of the T/C value.

### Use of test strips and evaluation of performance indicators

The test solution (100 μL) was pipetted into the lyophilized microwell with anti-aldicarb monoclonal antibody-labeled colloidal gold marker, which was slowly aspirated and fully mixed with the reagent in the microwell. Incubation occurred at 25°C for 3 min. Then, the liquid (100 μL) was dropped vertically into the sample hole of the test strip. Timing started when the liquid flowed, and the reaction was performed for 10 min. The color intensities of the T and C lines were read using a colloidal gold reader, and the T/C value was calculated.

The T/C value of the standard solution with aldicarb mass fractions of 0, 1.25, 2.5, 5, and 10 ng/mL was determined. Each mass fraction was tested 3 times in parallel, and the average value was calculated. A standard curve, with the aldicarb mass fraction on the abscissa and the T/C value on the ordinate, was drawn. The sensitivity and linear range of the prepared test strips were obtained from the standard curve. Twenty negative vegetable samples were analyzed, and the corresponding concentration of each sample T/C value was calculated using the standard curve. The detection limit was reported as the average concentration of 20 samples plus 3 times the standard deviation (31).

The positive samples prepared in section “Sample preparation” were measured. Six parallel tests were conducted with three batches of test strips at each level, and the addition recovery and intra-assay coefficient of variation were calculated using the actual measured value of each added mass fraction to judge the test strip accuracy and precision.

Twenty vegetable samples, including samples that were negative and exceeded the standard, were prepared by mixing vegetables with an aldicarb standard solution. The samples were randomly assigned a number between 1 and 20. The samples were tested by aldicarb test strips and an instrumental method to determine the coincidence rate of positive samples. The instrument detection method was carried out with reference to the method of China National Standard GB 23200.112-2018 (32).

The test strips and the lyophilized microporous reagent with anti-aldicarb monoclonal antibody-labeled colloidal gold were stored at 4^°^C, room temperature, and 37^°^C, respectively. The test strips were tested on days 1, 3, 6, 9, 12, and 15. The T/C values of cabbage samples with aldicarb concentrations of 0 and 30 μg/kg were investigated to evaluate the stability of the strip.

## Results and discussion

### Identification of artificial antigens

UV–Vis spectroscopy data were used to identify the coupling of the hapten to the carrier protein (Supplementary Figure 2). Obvious differences in the absorption wavelengths were observed among the conjugates and haptens. The hapten-to-protein molar ratios were estimated at 16.2 and 12.6 for haptens–KLHs and 5.2 and 4.1 for haptens–OVAs.

### Titer detection of aldicarb antiserum

After the fourth immunization, the blood of the mice was taken from the severed tail and examined using indirect ELISA. The absorbance at 450 nm (OD_450_) was measured using a microplate reader. The absorbance value of 0.8–1.2 was selected to calculate the inhibition rate, and the corresponding dilution multiple was used as the antiserum efficacy value. As shown in Table 1, the potency of the antiserum produced by the immunogen Hapten 1-KLH was 10,000, and the inhibition rate was 58.1%. This value was relatively high because Hapten 1 retained the molecular structure of aldicarb, which allowed recognition by immune active cells to the greatest extent and thus stimulation of the body to produce a specific immune response and antibodies with high specificity for the test object (33). Therefore, the antiserum produced using the immunogen Hapten 1-KLH was selected for the next experiment.

**TABLE 1:** Results for mouse antiserum.

Immunogen	Negative OD_450_	Positive OD_450_	Potency	Inhibition rate (%)*
Hapten 1-KLH	1.925	0.807	10,000	58.1
Hapten 2-KLH	1.582	0.874	3,000	44.7

*Inhibition rate = (negative OD_450_ – positive OD_450_)/negative OD_450_ × 100%. The concentrations of aldicarb used for determining the negative and positive OD_450_ were 0 and 30 μg/L, respectively.

### Immunological characteristics of anti-aldicarb monoclonal antibody

Three mice were immunized with the immunogen Hapten 1-KLH, and three positive cell lines were obtained after three immunizations, one booster immunization, and fusion cloning. The subtypes of the three cell lines were identified as MC-28, MC-54, and MC-65, all of which were IgG1. Analysis of the supernatant showed that the MC-54 cell line had the highest inhibition rate (Supplementary Table 2). Therefore, the MC-54 antibody was selected to develop colloidal gold immunochromatographic test strips in the subsequent experiments.

**FIGURE 2:**
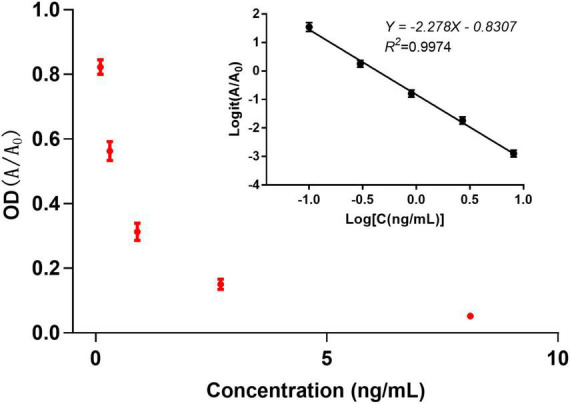
Indirect competitive inhibition curve of the anti-aldicarb monoclonal antibody.

The sensitivity of the anti-aldicarb monoclonal antibody was determined using icELISA. An indirect competitive inhibition curve, with the logarithm of the standard concentration as the abscissa and the Logit value of the percent absorbance at 450 nm (A/A_0_) as the ordinate, was drawn using GraphPad Prism 7.02 (Figure 2). The best fit had a correlation coefficient *R*^2^ of >0.99. The IC_50_ of the anti-aldicarb monoclonal antibody was 0.432 ng/mL, and the linear range (IC_20_–IC_80_) was 0.106–1.757 ng/mL.

Antibody specificity is the ability of the antibody to bind to a specific antigen in preference over antigen analogs. The cross-reactivity rate is often used as an evaluation standard because an inverse relationship exists between the cross-reactivity rate and the specificity of the antibody. In this study, we selected aldicarb sulfone, aldicarb sulfoxide, thiofanox, oxamyl, methomyl, metolcarb, isoprocarb, and carbofuran, with chemical structures similar to that of aldicarb, for the specificity analysis. The results showed that the monoclonal antibody was specific to aldicarb and had no significant cross-reactivity with aldicarb sulfone, aldicarb sulfoxide, thiofanox, oxamyl, methomyl, metolcarb, isoprocarb, or carbofuran (<1%) (Table 2).

**TABLE 2:** Cross-reactivity rates of anti-aldicarb monoclonal antibodies.

Compound	Chemical structure	Molecular formula	IC_50_ (μg/L)	Cross-reaction rate (%)
Aldicarb	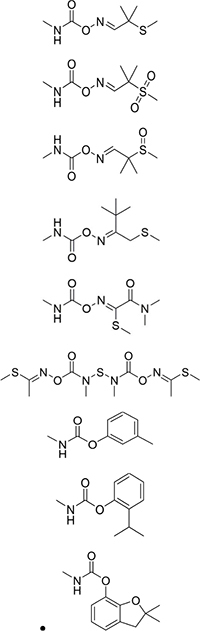	C_7_H_14_N_2_O_2_S	0.411	100
Aldicarb sulfone		C_7_H_14_N_2_O_4_S	>42	<0.978
Aldicarb sulfoxide		C_7_H_14_N_2_O_3_S	>42	<0.978
Thiofanox		C_9_H_18_N_2_O_2_S	>42	<0.978
Oxamyl		C_7_H_13_N_3_O_3_S	>42	<0.978
Methomyl		C_5_H_10_N_2_O_2_S	>42	<0.978
Metolcarb		C_9_H_11_NO_2_	>42	<0.978
Isoprocarb		C_11_H_15_NO_2_	>42	<0.978
Carbofuran		C_12_H_15_NO_3_	>42	<0.978

### Optimization of the test strip

Test strips were assembled with three different types of NC membranes (Milipore 90, Unisart CN 140, and Nupore 70) and used to detect the negative sample extract. The relative standard deviations (RSDs) of the T/C values were compared. Each NC membrane was tested 15 times. The RSD of Unisart CN 140 was lower than that of the other two membranes (Supplementary Table 4), and it was selected as the optimum NC membrane.

Similar experiments were performed to screen the working buffer for the sample pad. The lowest RSD was obtained with working buffer #5 (Supplementary Table 5). Therefore, the optimum working buffer for the sample pad was 0.02 M PBS (pH 7.4) containing 0.5% BSA, 0.05% Triton X-100, and 5.0% sucrose.

Next, sample pads prepared using different materials (nonwoven fabric, glass fiber, and whole blood filtration membrane) were treated with buffer #5 and used to assemble the test strip. Among these materials, the whole blood filtration membrane showed the fastest absorption rate for the sample solution (Supplementary Table 6). The glass fiber and nonwoven fabric had the same absorption rate, but the nonwoven fabric had the lowest RSD. Therefore, nonwoven fabric was selected as the best material for the sample pad.

### Evaluation of performance indicators of colloidal gold immunochromatographic test strips

**FIGURE 3:**
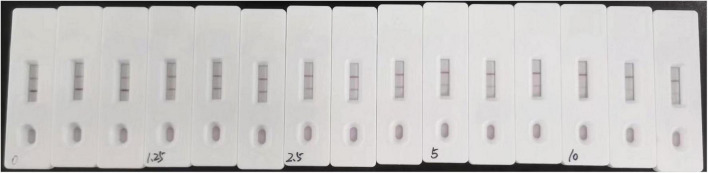
Colloidal gold immunochromatography assay for aldicarb in standard solution.

**FIGURE 4:**
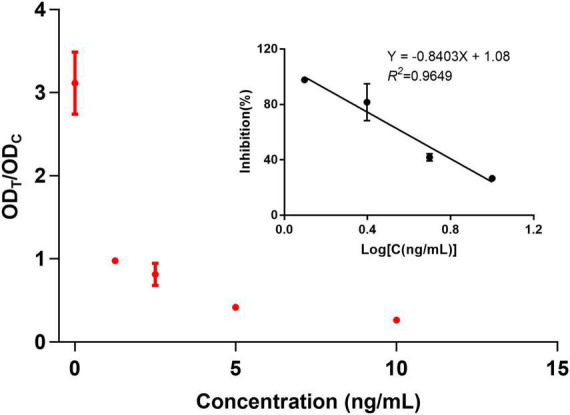
Standard curve of the colloidal gold immunochromatographic test strip.

The sensitivity of the assay was investigated with a series aldicarb standards. Figure 3 shows that the signal color on the test lines changed from strong (0 ng/mL) to weak and finally disappeared completely at 10 ng/mL aldicab. In the quantitative assay, the color intensities of the T and C lines were read using a colloidal gold reader, and the T/C value was calculated. Then, an indirect competitive inhibition curve, with the logarithm of the standard concentration as the abscissa and the T/C value from the T- and C-line color intensity of the colloidal gold readout (OD_T_/OD_C_) as the ordinate, was drawn using GraphPad Prism 7.02 (Figure 4). There was a good linear relationship between the T/C value of the test strip and the logarithm of the aldicarb concentration in the range of 1.25–10 ng/mL (Figure 4). The linear equation of the best fit was Y = –0.8403X + 1.08, and the correlation coefficient *R*^2^ was 0.9649. The T/C values of the samples were determined using the same method, and the aldicarb concentration in each sample was calculated using the standard curve.

**FIGURE 5:**
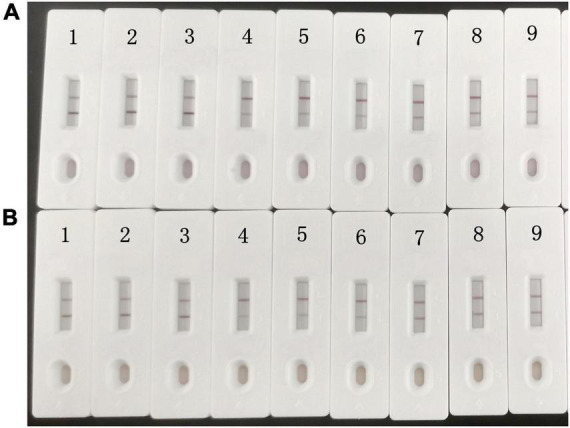
Colloidal gold immunochromatography assay for aldicarb in samples. Aldicarb concentration 1, 2, 3 = 0 μg/kg; 4, 5, 6 = 60 μg/kg; 7, 8, 9 = 30 μg/kg. **(A)** Cabbage. **(B)** Leek.

The detection limits of aldicarb in leek and cabbage samples were 26.11 and 28.39 μg/kg, respectively (Table 3). To ensure the accuracy and stability of the colloidal gold immunochromatographic method and to avoid false negatives, the detection limit of aldicarb in the vegetable samples was set to 30 μg/kg, which met the maximum residue limit specified in GB 2763 (7). The leek and cabbage samples were spiked with the aldicarb standard solution at final aldicard concentrations of 30 and 60 μg/kg. The spiked samples were analyzed with the test strip, and the results are summarized in Figure 5. Moreover, the aldicarb concentrations of the spiked samples were measured using a colloidal gold reader. As shown in Table 4, the average spiked recoveries of the leek and cabbage samples were 80.4%–110.5%, and the intra- and interassay relative standard deviations were <15%, indicating good accuracy and repeatability.

**TABLE 3:** Test strip detection limit determination results (μg/kg).

Leek				Cabbage			
Sample number	Measured value	Sample number	Measured value	Sample number	Measured value	Sample number	Measured value
1	5.6	11	12.2	1	13.6	11	2.1
2	1.8	12	6.1	2	4.7	12	9.5
3	0.2	13	5.5	3	0.8	13	6.7
4	11.3	14	9.4	4	2.6	14	21.6
5	2.7	15	7.8	5	11.9	15	0.0
6	4.9	16	22.4	6	0.0	16	1.7
7	20.6	17	1.5	7	6.1	17	24.6
8	0.0	18	3.9	8	15.2	18	8.8
9	8.2	19	14.8	9	7.3	19	4.5
10	3.5	20	6.6	10	5.8	20	10.6
Average value	7.45			Average value	7.90		
Standard deviation	6.22			Standard deviation	6.83		
Limit of detection	26.11			Limit of detection	28.39		

**TABLE 4:** Test strip accuracy and precision test results.

Sample	Add concentration (μg/kg)	Test strip batches	Recovery rate (%)	Intra-assay RSD (*n* = 6, %)	Average intra-assay RSD (%)	Inter-assay RSD (*n* = 3, %)
Leek	30	G-1	84.3/92.7/88.5/89.2/102.8/94.1	6.9	6.7	7.8
		G-2	96.4/82.9/90.3/91.6/85.2/85.6	5.7		
		G-3	94.7/110.5/95.6/89.7/95.5/103.5	7.6		
	60	G-1	100.5/108.3/92.7/103.1/101.9/105.8	5.3	6.7	8.6
		G-2	88.4/96.7/97.3/106.8/104.5/91.2	7.4		
		G-3	87.3/83.9/88.1/96.5/80.4/96.2	7.3		
Cabbage	30	G-1	85.6/93.1/86.4/85.9/100.4/81.7	7.6	7.9	9.5
		G-2	108.9/94.6/93.8/97.4/109.5/93.2	7.6		
		G-3	85.4/109.2/107.5/98.3/103.7/102.4	8.5		
	60	G-1	93.5/97.1/102.8/85.4/91.7/94.5	6.1	6.2	8.0
		G-2	105.1/99.6/106.2/89.5/100.3/108.6	6.7		
		G-3	88.2/82.9/92.3/93.7/84.4/95.1	5.7		

Among the 20 vegetable samples, eight samples were identified as positive by both the GB 23200.112-2018 standard method (32) and the test strip. Only sample 13 was identified as positive by the test strip (41.5 μg/kg) but negative by the instrumental method. Therefore, the test strip can accurately detect positive samples but with some false positives. This method could be applied to the detection and screening of aldicarb in vegetables, although the identification of positive samples needs to be confirmed by instrumental methods.

To evaluate the temperature stability, test strips were stored at 4°C, room temperature, and 37°C for 15 days. After storage, the test strips were used to analyze cabbage samples with aldicarb concentrations of 0 and 30 μg/kg. The T/C values obtained showed no significant changes (Supplementary Figure 3).

## Conclusion

Aldicarb is a small molecule without immunogenicity and must be conjugated with carrier proteins to induce an appropriate immune response in animals. To synthesize complete antigens, haptens must be rationally designed to obtain high specificity (34). Two haptens, Hapten 1 and Hapten 2, with spacers of different carbon chain lengths were designed and synthesized using 2-methyl-2-(methylsulfanyl)propanaldoxime as the raw material. Hapten 1 retained more of the aldicarb molecular structure than Hapten 2, and from an analysis of the length of the introduced side chain, the distance of a chain of three carbon atoms ending in the -COOH group on the aldicarb molecule satisfied the distance for minimal interaction between each carrier protein. Effective coupling of aldicarb is required to expose the epitope of the aldicarb molecule, thus improving the specificity of the antibody (35). After immunizing the mice, we successfully obtained a highly specific and highly sensitive anti-aldicarb monoclonal antibody with an IC_50_ of 0.432 ng/mL and a linear range of 0.106–1.757 ng/mL, which showed no cross-reactivity with other structural analogs. Colloidal gold immunochromatography based on the prepared monoclonal antibody was demonstrated to be a rapid method for the detection of aldicarb residues in vegetables, with a detection limit of 30 μg/kg and a recovery rate of 80.4–110.5%. Moreover, the relative standard deviation between batches was less than 15%. Compared with instrumental analysis methods, colloidal gold immunochromatography has the advantages of simple operation, rapid detection, and low cost. However, owing to the technical limitations of colloidal gold immunochromatography, problems such as false positives may occur, and thus positive samples need to be confirmed through instrumental methods. Nevertheless, as a rapid screening method, this method can be widely used for the on-site screening and detection of aldicarb residues in vegetables and has important economic and social value.

## Data availability statement

The original contributions presented in this study are included in the article/Supplementary material, further inquiries can be directed to the corresponding author.

## Ethics statement

This animal study was reviewed and approved by the Beijing Kwinbon Biotechnology Co., Ltd. Written informed consent was obtained from the owners for the participation of their animals in this study.

## Author contributions

MD and YW: conception and design of the study. HSh and YW: methodology. HSh, JL, HSu, and YZ: data curation. HC, XW, and KH: data statistical analysis. GH, XC, and GL: validation. TC: writing the first draft of the manuscript. All authors contributed to manuscript revision, read, and approved the submitted version.

## References

[1] Sajwan RK Lakshmi GBVS Solanki PR. Fluorescence tuning behavior of carbon quantum dots with gold nanoparticles *via* novel intercalation effect of aldicarb. *Food Chem.* (2020) 340:127835. 10.1016/j.foodchem.2020.127835 33002825

[2] Küster E Altenburger R. Suborganismic and organismic effects of aldicarb and its metabolite aldicarb-sulfoxide to the zebrafish embryo (*Danio rerio*). *Chemosphere.* (2007) 68:751–60. 10.1016/j.chemosphere.2006.12.093 17292441

[3] Fisher IJ Phillips PJ Bayraktar BN Chen S McCarthy BA Sandstrom MW Pesticides and their degradates in groundwater reflect past use and current management strategies, Long Island, New York, USA. *Sci Tot Environ.* (2020) 752:141895. 10.1016/j.scitotenv.2020.141895 32892047

[4] Li YB Qin GF He FR Zou K Zuo B Liu R Investigation and analysis of pesticide residues in edible fungi produced in the mid-western region of China. *Food Control.* (2022) 136:108857. 10.1016/j.foodcont.2022.108857

[5] Boucaud-Maitre D Ranabourg MO Sinno-Tellier S Puskarczyk E Pineau X Kammerer M Human exposure to banned pesticides reported to the French Poison Control Centers: 2012-2016. *Environ Toxicol Pharmacol.* (2019) 69:51–6. 10.1016/j.etap.2019.03.017 30953934

[6] Somashekar KM Mahima MR Manjunath KC. Contamination of water sources in Mysore City by pesticide residues and plasticizer – a cause of health concern. *Aquatic Proc.* (2015) 4:1181–8. 10.1016/j.aqpro.2015.02.150

[7] GB 2763-2021. *National Food Safety Standard—Maximum Residue Limits For Pesticides In Food.* Beijing: China Agricultural Press (2021).

[8] Dai XL Luan Y Wang XY Hua S Hu C. Gas chromatographic determination of aldicarb and its metabolites in urine. *J Chromatogr A.* (1991) 542:526–30. 10.1016/S0021-9673(01)88788-4 1880190

[9] Nunes GS Alonso RM Ribeiro ML Barceló D. Determination of aldicarb, aldicarb sulfoxide and aldicarb sulfone in some fruits and vegetables using high-performance liquid chromatography-atmospheric pressure chemical ionization mass spectrometry. *J Chromatogr A.* (2000) 888:113–20. 10.1016/s0021-9673(00)00553-7 10949478

[10] Totti S Fernández M Ghini S Picó Y Fini F Mañes J Application of matrix solid phase dispersion to the determination of imidacloprid, carbaryl, aldicarb, and their main metabolites in honeybees by liquid chromatography-mass spectrometry detection. *Talanta.* (2006) 69:724–9. 10.1016/j.talanta.2005.11.012 18970629

[11] Wang YL Xu JL Qiu YL. Highly specific monoclonal antibody and sensitive quantum dot beads-based fluorescence immunochromatographic test strip for tebuconazole assay in agricultural products. *J Agric Food Chem.* (2019) 67:9096–103. 10.1021/acs.jafc.9b02832 31356079 PMC7069222

[12] Esteve-Turrillas FA Mercader JV Agullo C Abad-Somovilla A Abad-Fuentes A. Highly sensitive monoclonal antibody-based immunoassays for boscalid analysis in strawberries. *Food Chem.* (2018) 267:2–9. 10.1016/j.foodchem.2017.06.013 29934157

[13] Brady JF Fleeker JR Wilson RA. Enzyme immunoassay for aldicarb. *Am Chem Soc.* (1988) 1021:262–84. 10.1021/bk-1988-0382.ch021

[14] Siew LK Winger LA Spoors JA Dessi JL Jennens L Self CH Monoclonal antibodies useful in sensitive immunoassays for aldicarb in either laboratory or the field. *Int J Environ Anal Chem.* (2003) 83:417–26. 10.1080/0306731031000099819

[15] Zhang YF Gao ZX Zhang QM Dai SG. A new hapten for immunoassay of aldicarb. *Chin Chem Lett.* (2006) 17:1021–4.

[16] Yao LJ. *Development of rapid immunoassay of imidacloprid, acetamiprid, carbofuran and aldicarb.* Jiangsu: Jiangnan University (2017).

[17] Zhang M Yan H Liu BF. Toxicity and bio-monitoring of dichloromethane: A review of recent studies. *J Environ Health.* (2015) 32:1108–12.

[18] Liu LQ Suryoprabowo S Zheng QK Song S Kuang H. Rapid detection of aldicarb in cucumber with animmunochromatographic test strip. *Food Agric Immunol.* (2017) 28:427–38. 10.1080/09540105.2017.1293015

[19] Weltzien HU Moulon C Martin S Padovan E Hartmann U Kohler J T cell immune responses to haptens. Structural models for allergic and autoimmune reactions. *Toxicology.* (1996) 107:141–51. 10.1016/0300-483x(95)03253-c 8599173

[20] Kim YJ Cho YA Lee HS Lee YT. Investigation of the effect of hapten heterology on immunoassay sensitivity and development of an enzyme-linked immunosorbent assay for the organophosphorus insecticide fenthion. *Anal Chim Acta.* (2003) 494:29–40. 10.1016/j.aca.2003.07.003

[21] Kim YJ Cho YA Lee HS Lee YT Gee SJ Hammock BD Synthesis of haptens for immunoassay of organophosphorus pesticides and effect of heterology in hapten spacer arm length on immunoassay sensitivity. *Anal Chim Acta.* (2003) 475:85–96. 10.1016/S0003-2670(02)01037-1

[22] Mari GM Li HF Dong BL Yang H Talpur A Mi J Hapten synthesis, monoclonal antibody production and immunoassay development for direct detection of 4-hydroxybenzehydrazide in chicken, the metabolite of nifuroxazide. *Food Chem.* (2021) 355:129598. 10.1016/j.foodchem.2021.129598 33765482

[23] Esteve-Turrillas FA Meracde JV Agullo C Abad-Somovilla A Abad-Fuentes A. Site-heterologous haptens and competitive monoclonal antibody-based immunoassays for pyrimethanil residue analysis in foodstuffs. *LWT Food Sci Technol.* (2015) 63:604–11. 10.1016/j.lwt.2015.03.074

[24] Lee JK Ahn KC Stoutamire DW Gee SJ Hammock BD. Development of an enzyme-linked immunosorbent assay for the detection of the organophosphorus insecticide acephate. *J Agric Food Chem.* (2003) 51:3695–703. 10.1021/jf021020i 12797729

[25] Li YQ. Study on immunoassay of quinclorac and bensulfuron methyl. *Chin Acad Agric Sci Thesis.* (2021). 10.27630/d.cnki.gznky.2021.000732

[26] Shen H Li C Sun H Chen W Chen B Yi Y Generation and characterization of an anti-diclazuril monoclonal antibody and development of a diagnostic ELISA for poultry. *Front Nutr.* (2022) 9:910876. 10.3389/fnut.2022.910876 35651507 PMC9149080

[27] Kuang H Xing C Hao C Liu L Wang L Xu C Rapid and highly sensitive detection of lead ions in drinking water based on a strip immunosensor. *Sensors.* (2013) 13:4214–24. 10.3390/s130404214 23539028 PMC3673080

[28] Li PW Zhou Q Wang T Zhou H Zhang W Ding X Development of an enzyme-linked immunosorbent assay method specific for the detection of G-group aflatoxins. *Toxins.* (2016) 8:1–11. 10.3390/toxins8010005 26729164 PMC4728527

[29] Putalun W Morinaga O Tanaka H. Development of a one-step immunochromatographic strip test for the detection of sennosides A and B. *Phytochem Anal.* (2004) 15:112–6. 10.1002/pca.752 15116942

[30] Liu X Xiang JJ Tang Y Zhang XL Fu QQ Zou JH Colloidal gold nanoparticle probe-based immunochromatographic assay for the rapid detection of chromium ions in water and serum samples. *Anal Chim Acta.* (2012) 745:99–105. 10.1016/j.aca.2012.06.029 22938612 PMC3468954

[31] SN/T 2775-2011. *Methods for the evaluation of commercial test kits for food testing purpose*. China’s General Administration of Quality Supervision, Inspection and Quarantine (2011).

[32] GB 23200.112-2018. *National Food Safety Standard Determination Of 9 Carbamate Pesticides And Metabolites Residues In Foods Of Plant Origin Liquid Chromatography Post Column Derivatization Method.* Beijing: China Agricultural Press (2021).

[33] Goodrow MH Hammock BD. Hapten design for compound-selective antibodies: ELISAs for environmentally deleterious small molecules. *Anal Chim Acta.* (1998) 376:83–91. 10.1016/S0003-2670(98)00433-4

[34] Gefen T Vaya J Khatib S Rapoport I Lupo M Barnea E The effect of haptens on protein-carrier immunogenicity. *Immunology.* (2015) 144:116–26. 10.1111/imm.12356 25041614 PMC4264915

[35] Shelver WL Smith DJ. Enzyme-linked immunosorbent assay development for the beta-adrenergic agonist zilpaterol. *Agric Food Chem.* (2004) 52:2159–66. 10.1021/jf049919i 15080615

