# Supplementary material for: Hapten designs based on aldicarb for the development of a colloidal gold immunochromatographic quantitative test strip
Source: Front Nutr. 2022 Aug 23;9:976284. doi: 10.3389/fnut.2022.976284 (PMC9446148; doi:10.3389/fnut.2022.976284)
Supplement: Supplementary file 1 [file Data_Sheet_1.docx]

Supplementary Material

# Parameters for LC analysis

a) Column: C_8_, 250 mm×4.6 mm×5 μm.

b) Column temperature: 42 °C.

c) FLD: *λ*_ex_＝330 nm, *λ*_em_＝465 nm.

d) Mobile phase and gradient elution conditions are shown in Table S1.

**Table S1. Mobile phase and gradient elution conditions (*V*_A_**+ ***V*_B_).**

| Time  min | Flow  mL/min | Mobile phase (water)*V*_A_ | Mobile phase (methanol)*V*_B_ |
| --- | --- | --- | --- |
| 0.00 | 1.0 | 85 | 15 |
| 2.00 | 1.0 | 75 | 25 |
| 6.50 | 1.0 | 75 | 25 |
| 10.50 | 1.0 | 60 | 40 |
| 28.00 | 1.0 | 60 | 40 |
| 33.00 | 1.0 | 20 | 80 |
| 35.00 | 1.0 | 20 | 80 |
| 35.10 | 1.0 | 0 | 100 |
| 37.00 | 1.0 | 0 | 100 |
| 37.10 | 1.0 | 85 | 15 |

e) Post-column derivatization: 0.05 mol/L NaOH at 0.3 mL/min, OPA reagent at 0.3 mL/min,hydrolysis temperature of 100 °C, andderivatization temperature of room temperature.

f) Injection volume: 10 μL.

# Screening of hybridoma cell lines

**Table S2. Screening experiment of hybridoma cell lines.**

| Hybridoma cell lines | Negative OD_450_ | Positive OD_450_ | Potency | Inhibition rate(%)^*^ |
| --- | --- | --- | --- | --- |
| MC-28 | 2.235 | 0.406 | 90000 | 81.8 |
| MC-54 | 2.161 | 0.205 | 90000 | 90.5 |
| MC-65 | 2.607 | 0.698 | 90000 | 73.2 |

^*^: Inhibition rate = (negative OD_450_ － positive OD_450_)/negative OD_450_×100%. The concentrations of aldicarb used for determining the negative and positive OD_450_were 0 and 3 μg/L, respectively.

# Thermal stability analysisof the antibody

The monoclonal antibody against aldicarb was stored at 37 °C. The OD values of standard solutions of aldicarb with a series of concentrations were measured by icELISA on days 0, 1, 4, and 7. The OD value of the standard solution showed no significant change after the antibody was stored at 37 °C for a week (Table S3), and the stability was good.

**Table S3. Thermal stability of the aldicarb antibody.**

| Concentration of aldicarb standard (ng/mL) | Day 0 | Day 1 | Day 4 | Day 7 | Day 4 descending rate | Day 7 descending rate |
| --- | --- | --- | --- | --- | --- | --- |
| 0 | 2.002 | 1.963 | 1.804 | 1.755 | 9.9% | 12.3% |
| 0.1 | 1.580 | 1.451 | 1.367 | 1.329 | 13.5% | 15.9% |
| 0.3 | 1.130 | 1.026 | 0.972 | 0.891 | 14.0% | 21.1% |
| 0.9 | 0.603 | 0.557 | 0.526 | 0.483 | 12.8% | 19.9% |
| 2.7 | 0.286 | 0.278 | 0.245 | 0.224 | 14.3% | 21.7% |
| 8.1 | 0.112 | 0.109 | 0.093 | 0.088 | 17.0% | 21.4% |

# Optimization of the test strip

**Table S4. Screening of NC membranes.**

| The type of NC membrane | Average T/C value (*n* = 15) | RSD (%, *n* = 15) |
| --- | --- | --- |
| Milipore 90 | 1.983 | 9.5 |
| Unisart CN 140 | 2.227 | 4.9 |
| Nupore 70 | 2.061 | 11.6 |

**Table S5. Screening of working buffers for the sample pad.**

| Number | Component | Average T/C value  (*n* = 15) | RSD  (%, *n* = 15) |
| --- | --- | --- | --- |
| 1# | PB+0.5% BSA | 2.224 | 12.8 |
| 2# | PB+0.5% BSA+2.5%sucrose | 1.965 | 10.4 |
| 3# | PB+0.5% BSA+0.05% Triton X-100 | 2.101 | 13.7 |
| 4# | PB+0.5% BSA+0.1% Triton X-100+2.5%sucrose | 2.087 | 9.9 |
| 5# | PB+0.5% BSA+0.05% Triton X-100+5.0%sucrose | 2.316 | 5.1 |

**Table S6. Screening of the sample pad.**

| Material | Time required to pass through NC membrane/s | Average T/C value  (*n* = 15) | RSD  (%, *n* = 15) |
| --- | --- | --- | --- |
| Whole blood filtration membrane | 33 | 2.142 | 11.5 |
| Glass fibre | 40 | 1.987 | 10.6 |
| Non-woven fabric | 42 | 2.273 | 4.3 |

# Supplementary Figures

**(A)
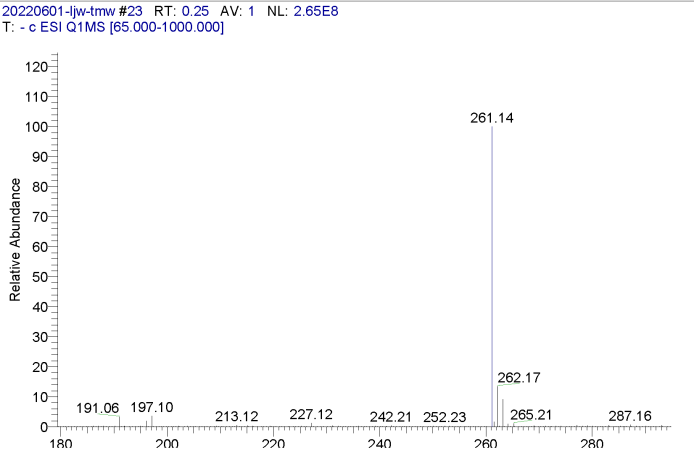
(B)
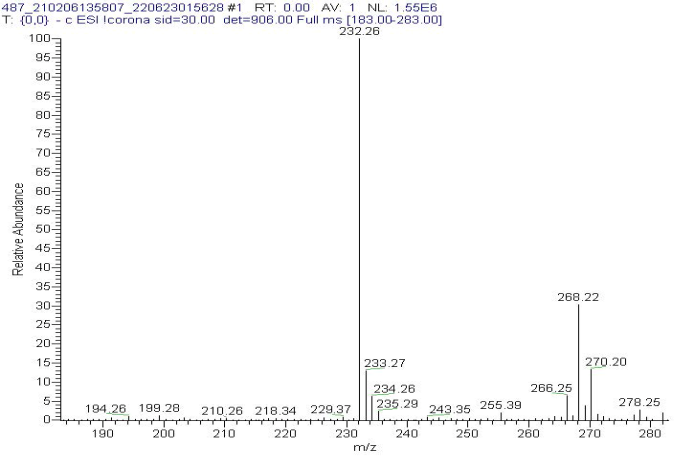
**

**(C)
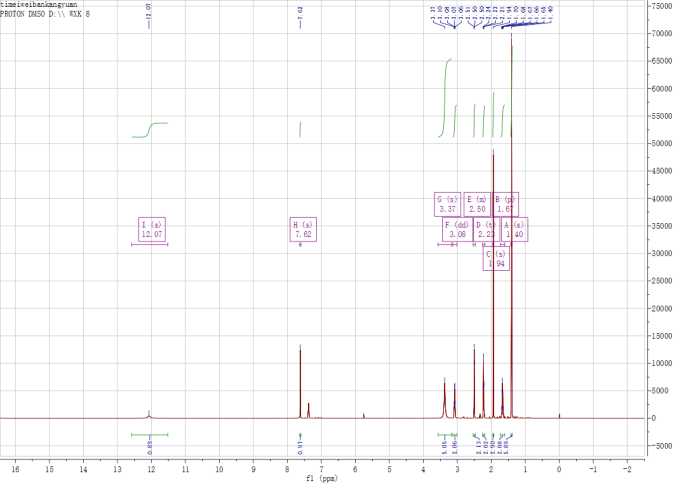
(D)
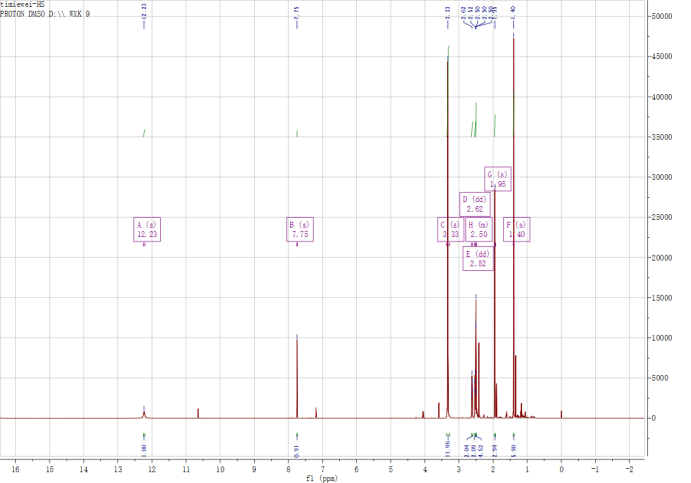
**

**Fig.S1. (A) Mass spectrum of Hapten 1, (B) mass spectrum of Hapten 2, (C) NMR spectrum (^1^H) of Hapten 1, and (D) NMR spectrum (^1^H) of Hapten 2.**

**(A)**
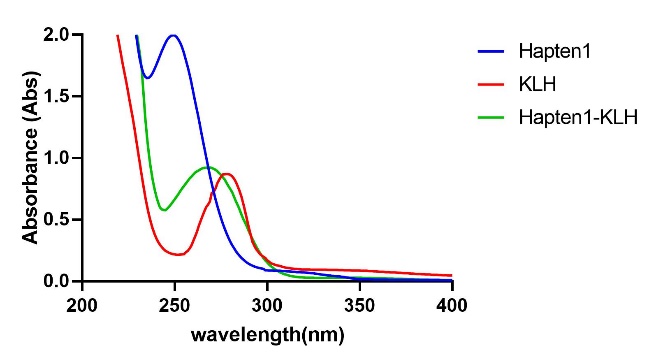
 **(B)**
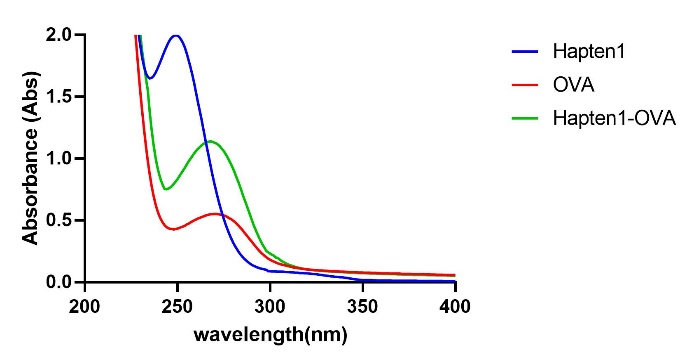


**(C)**
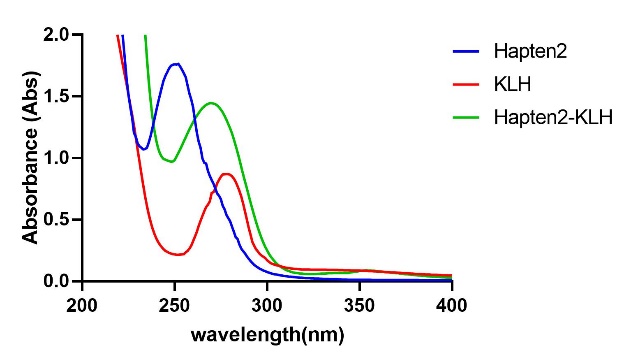
 **(D)**
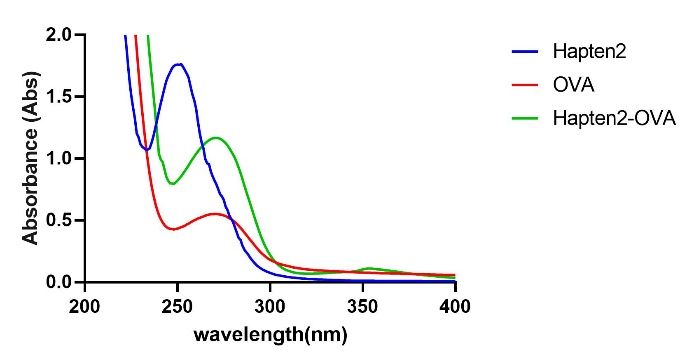


**Fig.S2. UV absorption spectra of haptens, carrier proteins, and conjugates.**

**
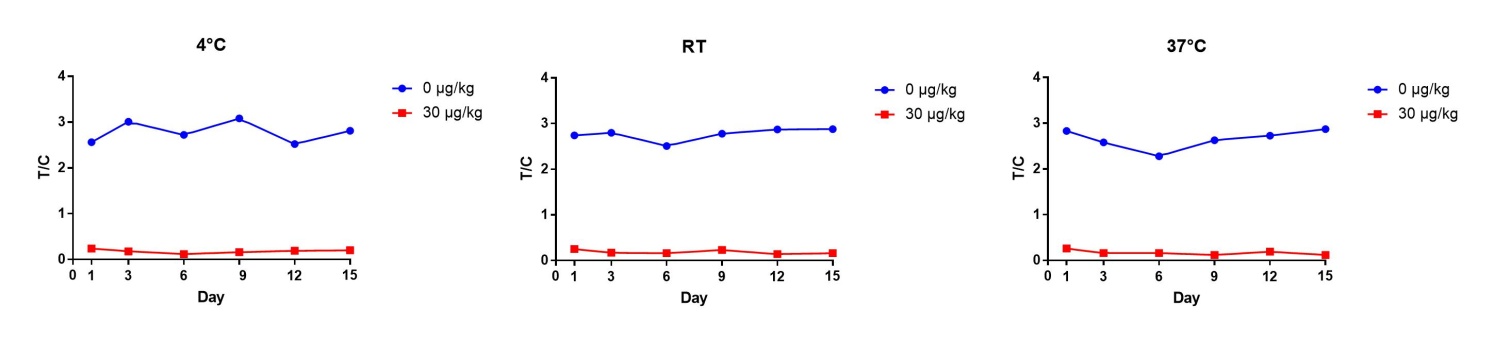
**

**Fig. S3. Stability of the test strip.**
